# Elastic enhancer network tunes equilibrium thermodynamics of liquid liquid phase separation in super enhancers

**DOI:** 10.1016/j.isci.2026.115152

**Published:** 2026-03-04

**Authors:** Tinghe Guo, Nan Zhang, Yannan Li, Shaoqian Hao, Junjie Liu, Lirong Zhang

**Affiliations:** 1 School of Physical Science and Technology, Key Laboratory of Biophysics and Bioinformatics of Inner Mongolia Autonomous Region, Inner Mongolia University, Hohhot 010021, China

**Keywords:** Biomolecules, Molecular biology, Biophysics

## Abstract

Super-enhancers (SEs) are clusters of enhancers that drive higher transcriptional output than typical enhancers (TEs) and regulate key genes central to cell type-specific gene expression programs. Liquid-liquid phase separation (LLPS) mediates the formation of SE-associated droplets with distinct functional properties. Here, we develop a theoretical framework in which chromatin architecture mechanically constrains LLPS droplet growth. Quantitative modeling within a binary phase separation system shows that the growth increment scales as $\Delta R\propto k^{-1/2}$, where k denotes the interaction strength between enhancer elements, thereby limiting droplet growth and supporting SE size homeostasis in the nucleus. Model predictions agree quantitatively with experiments and indicate that mechanical constraints regulate the critical concentration and miscibility of SE-associated droplets. Mechanical constraints may potentially regulate local concentration by altering the osmotic pressure difference across the droplet interface, which may in turn modulate transcriptional activity.

## Highlights

• Super-enhancer chromatin architecture limits LLPS droplet size
• Droplet growth shows a power-law dependence on interaction strength
• Mechanical constraint tunes LLPS critical concentration and miscibility

## Graphical abstract

**Figure:**
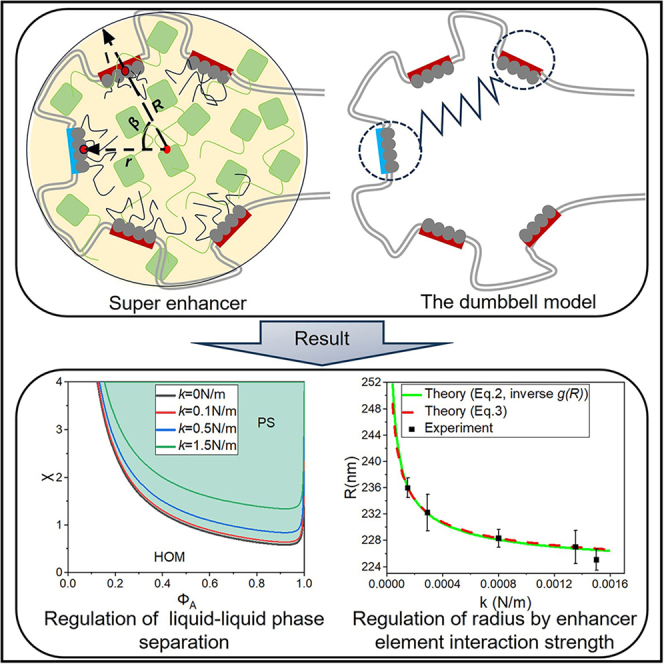

## Introduction

Eukaryotic cells achieve functional regulation through compartmentalization via both membrane-bound organelles (MBOs) and membraneless organelles (MLOs).^1^ The latter were first observed by Felice Fontana during the interphase of cell division in the 1770s,^2^ and later termed “nucleoli” by Rudolf Wagner in the 1830s.^3^ MLOs are widely distributed in eukaryotes^4^ and mediate critical functions such as gene transcription, expression, and regulation.^5^^,^^6^ Unlike MBOs, MLO components dynamically assemble via liquid-liquid phase separation in response to microenvironmental cues, enabling rapid adaptation to cellular states.^6^

Liquid-liquid phase separation (LLPS) describes a phenomenon wherein multicomponent solutions spontaneously separate into two immiscible liquid phases under specific conditions. This process occurs when intermolecular interactions induce a decrease in the system’s free energy.^7^ In biological systems, LLPS is predominantly driven by multivalent interactions,^8^ serving as a fundamental mechanism for the formation of MLOs.^9^ Biomolecular enrichment via LLPS generates condensed-phase droplets with 10−−100-fold concentration gradients relative to the dilute phase,^2^ functionally compartmentalizing biochemical processes. These LLPS droplets not only achieve high biomolecular concentrations but also exhibit reduced molecular mobility compared to the bulk solution. Such physical characteristics have been shown to enhance biochemical reaction kinetics through both spatial confinement and selection effects.^8^

In the 1980s, Banerji et al. identified enhancers as *cis*-regulatory elements capable of amplifying target gene transcription.^10^ Three decades later, the Young team discovered clustered enhancer domains in mouse embryonic stem cells, termed “super-enhancers” (SEs), which exhibited distinct structural and functional properties compared to typical enhancers (TEs).^11^ SEs are capable of recruiting high densities of transcription factors and cofactors, thereby driving the expression of genes critical for cell identity determination.^12^ Further studies have revealed that a single SE can also interact with multiple promoters through chromatin looping structures to coordinately regulate the expression of multiple target genes within a gene cluster.^13^ As a result, compared to TEs, SEs exhibit significantly higher transcriptional activation efficiency.^14^^,^^15^^,^^16^^,^^17^ Dysregulation of SEs is closely associated with the pathogenesis and progression of various diseases. For example, in Alzheimer’s disease research, five single-nucleotide polymorphism (SNP) loci located within SEs in brain tissue have been identified as disease-associated, accounting for approximately 19% of known Alzheimer’s disease-related SNPs.^18^^,^^19^ In cancer research, evidence indicates that cancer cells can promote tumorigenesis by constructing SEs that specifically drive the expression of oncogenes.^15^^,^^20^ A 2022 study discovered a tumor-specific SE within the gene desert region of the c-myc oncogene.^21^ A 2024 study on lung adenocarcinoma (LUAD) further revealed that cancer-specific SEs target genes enriched in tumor driver genes and related signaling pathways, whereas SEs in normal cells primarily regulate immune-related genes.^22^ This aberrant activation of target genes may be linked to structural alterations in SEs.^23^ Notably, in cancer, SEs can either drive the expression of oncogenes (e.g., MYC) or activate tumor suppressor genes (e.g., TP53), depending on the cellular context.^24^^,^^25^ As a result, SEs are increasingly regarded as promising therapeutic targets. For instance, in acute myeloid leukemia (AML)^26^ and primary effusion lymphoma (PEL),^27^^,^^28^ researchers have attempted to upregulate the activity of specific SEs to activate tumor suppressor and survival-related genes, thereby achieving therapeutic effects. Conversely, in pancreatic ductal adenocarcinoma (PDAC)^29^ and malignant glioblastoma,^30^ suppression of SE-associated gene expression has been employed to inhibit cancer cell growth. Collectively, these studies demonstrate the crucial role of SEs in disease regulation^14^^,^^15^^,^^31^^,^^32^ and highlight their broad potential for therapeutic intervention.

These SEs feature tightly spaced enhancer elements (approximately 12.5 kb intervals), flanked by CTCF binding sites and delimited by boundary elements.^33^ The teams led by Denes Hnisz and Benjamin R. independently demonstrated the presence of LLPS structures within SEs through computational modeling and experimental approaches, respectively.^34^^,^^35^ They identified this phase-separated architecture as a key mechanism underlying the higher sensitivity of SE-regulated gene expression to transcriptional inhibitors compared to that regulated by TEs.^34^^,^^35^ Additionally, ChIP-seq analysis^34^ revealed that p300, transcription factors (TFs), H3K27ac, BRD4, and Mediator complex components exhibited a high degree of spatial overlap, suggesting that SE formation and transcriptional activation occur within a shared LLPS-driven compartment.

In contrast to canonical LLPS observed in biological systems, the formation of LLPS structures within SEs necessitates an open chromatin compartment with regulated spatial dimensions to accommodate their volumetric requirements. Through investigations of elastic network effects, elastic networks have been shown to substantially suppress both LLPS nucleation and growth.^36^ Notably, while LLPS droplets in eukaryotic cells typically exhibit characteristic sizes of 0.2–2 μm,^37^ LLPS droplets linked to SEs display markedly reduced dimensions and heightened transient dynamics.^3^

The findings suggest that the chromatin architecture associated with SEs not only provides a physical confinement for the formation of internal LLPS droplets but may also directly regulate these droplets through the exertion of mechanical constraints, thereby influencing gene expression. However, current research has primarily focused on the transcriptional regulatory functions of SEs and the structure and dynamics of LLPS droplets within them, as exemplified by the seminal model proposed by Hnisz et al., which explains how SEs undergo LLPS through multivalent interactions of TFs, leading to the formation of LLPS droplets that efficiently orchestrate gene expression.^34^ There remains a lack of in-depth investigation into the functions of SE-associated chromatin beyond its role in encoding genetic information. A crucial scientific question that remains unresolved is how SE-associated chromatin precisely controls the growth processes of LLPS, particularly within the highly crowded nuclear milieu.

**Figure 1:**
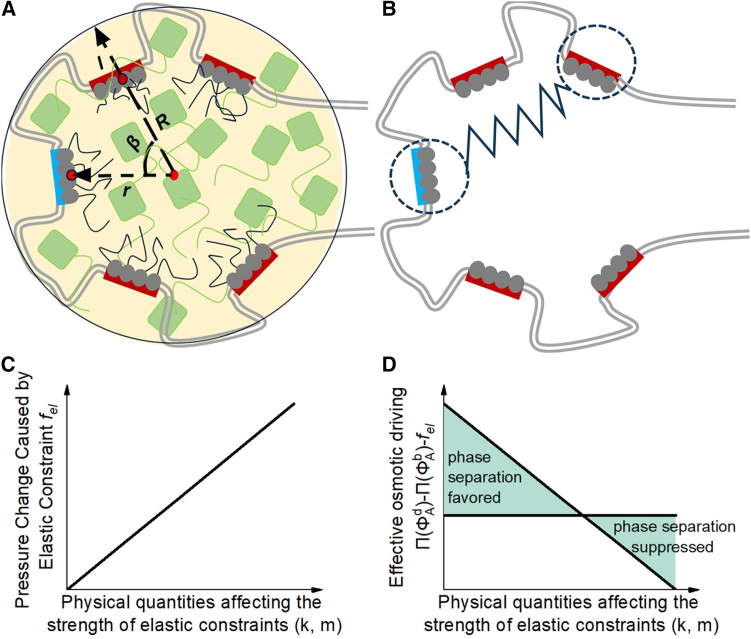
Schematic diagrams of super-enhancer (SE) architecture and constraint models (A) SE architecture: rectangles represent enhancer elements (blue: classical enhancers; red: facilitator elements), squares denote coactivators, ovals indicate transcription factors, and wavy patterns symbolize intrinsically disordered regions (IDRs). Double solid lines delineate intra-SE chromatin architecture. Key parameters include R (radius of the droplet), r (distance of an enhancer element from the droplet center), β (central angle between two enhancer elements), and l (penetration depth of enhancer elements into the droplet). (B) The dumbbell model: dashed circles represent enhancer elements, with a spring illustrating the interaction between classical enhancer and facilitator elements. (C) The effect of enhancer element interaction strength k and size m on the pressure change induced by elastic constraints. (D) The dependence of the effective osmotic driving on enhancer element interaction strength k and element size m.

Thus, it is hypothesized that SEs undergo chromatin-mediated elastic confinement to adapt to the densely crowded nuclear environment. Within SE architectures, we identify intra-SE chromatin as a mechanical constraint regulating LLPS dynamics. We define the transcription factor binding sites (TFBSs) and their flanking sequences as the enhancer element of size m, which scales the relative size of the enhancer element, and the effective stiffness of the protein-mediated chromatin loop is abstracted as the interaction strength k between enhancer elements. This parameter k can serve as a quantitative measure metric for chromatin-imposed confinement. Our free energy model systematically integrates four essential components: mixing entropy of the chromatin-polymer system, Flory-Huggins-type phase separation energy, interfacial tension of LLPS droplets, and enhancer element interaction energies. Focused analysis reveals how two intrinsic SE parameters—enhancer element size m and interaction strength k—jointly dictate LLPS droplet formation and spatial confinement within SE domains. These theoretical predictions show strong agreement with experimental observations of SE-associated LLPS droplets. As shown in Figures 1C and 1D, mechanical confinement inhibits LLPS. Further investigation has revealed that mechanical confinement elevates the critical concentration for LLPS and enhances miscibility. Existing studies indicate that the function of SEs generally aligns with an additive model, while the activity of individual enhancers is directly regulated by the concentration of TFs.^38^^,^^39^ The model proposed in this study further demonstrates that mechanical confinement can increase the saturation concentration within LLPS droplets by modulating the osmotic pressure difference between the interior and exterior of the droplets. This offers a mechanistic explanation for how mechanical confinement may potentially enhance the transcriptional activity of SEs. This insight provides a theoretical perspective for investigating the regulation of SEs expression from a mechanical standpoint.

## Results

### Key model equations

To quantitatively describe the regulatory effect of SE-associated chromatin architecture on the size of LLPS droplets, we introduce an additional energy contribution arising from deformations of the peripheral chromatin structure surrounding super enhancers into a binary-mixture free-energy framework. By idealizing the effective rigidity of protein-mediated chromatin loops as an interaction strength between enhancer elements (Figure 1B), and adopting a linear (Hookean-type) elasticity assumption, we obtain an analytical expression for the elastic energy density.

(Equation 1) $$f_{\text{el}}=\overset{n-1}{\underset{y=1}{\sum }}\frac{1}{2}\frac{km\left(1-\cos\beta \right)}{\Delta h}\frac{{\left(2\left(n+\pi \right)R-L\right)}^{3}}{4\pi n^{2}\left(n+\pi \right)R^{3}},$$

where β=2πy/n, y represents the relative displacement between two enhancer elements, m represents the element sizes, n denotes the total number of elements, L is the length of intra-SE chromatin, R corresponds to the radius of the LLPS droplet (the associated geometric definition is illustrated in Figure 1A), k is the enhancer-facilitator interaction strength, and Δh is defined as the microscopic effective width (substantially smaller than the droplet size) over which the SE-associated chromatin structure acts. It is set to unity for simplicity in subsequent discussions. These parameters collectively define the chromatin-mediated mechanical constraints.

Under thermodynamic equilibrium, the osmotic pressure difference between the bulk and droplet phases is balanced by the surface-tension term together with an elastic contribution arising from deformations of the SE-associated chromatin architecture. To discuss the relationship between the size of LLPS droplets and the free energy density, we analyze the derivative of the elastic free energy density $f_{\text{el}}$ with respect to R. This reveals the competition between surface tension and chromatin-mediated elastic forces:

(Equation 2) $$\begin{array}{cc} \frac{3\gamma }{R} & =R\frac{df_{\text{el}}}{dR}\\ & =\overset{n-1}{\underset{y=1}{\sum }}\frac{1}{2}\frac{km\left(1-\cos\beta \right)}{\Delta h}\frac{3L{\left(L-2\left(n+\pi \right)R\right)}^{2}}{4\pi n^{2}\left(n+\pi \right)R^{4}}R\\ & =g\left(R\right), \end{array}$$

where β=2πy/n. This equality demonstrates how surface tension counterbalances elastic forces to determine droplet size. The function g(R) encapsulates enhancer element interactions through the parameters k, m, n, and L defined in Equation 1.

In soft-matter physics, the coil size of an ideal Gaussian chain scales as a power law with the elastic constant.^40^^,^^41^ Accordingly, we derive an expression for the overall equilibrium radius of the SE-associated droplet as follows:

(Equation 3) $$R=R_{0}+\Delta R=\frac{L}{2n+2\pi }+\frac{n+\pi }{\pi }\cdot {\left(\frac{k_{\text{B}}T}{k}\right)}^{\frac{1}{2}}.$$

where $\Delta R_{\max}$ denotes the maximum change in the droplet radius, $k_{\text{B}}$ is the Boltzmann constant, T denotes the temperature, $R_{0}$ is the initial droplet radius, n denotes the total number of elements, and L is the intra-SE chromatin length. Detailed derivations of (Equation 1), (Equation 2), (Equation 3), together with the underlying assumptions, are provided in STAR Methods.

### Enhancer interaction strength tunes droplet radius and LLPS phase diagram

**Figure 2:**
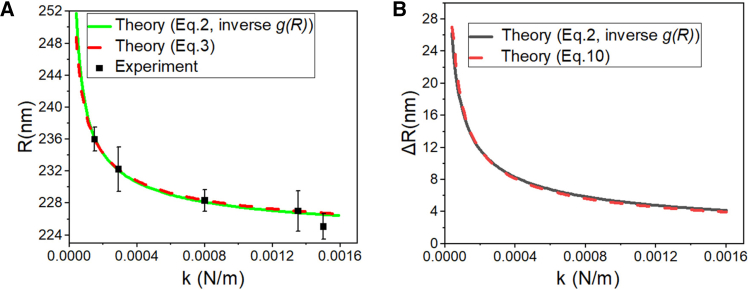
Theoretical and experimental scaling of SE radius with enhancer interaction strength in mouse embryonic stem cells (mESCs) (A) Dependence of SE radius R on enhancer interaction strength k. Green solid line: inverse structural correlation function $g{\left(R\right)}^{-1}$; red dashed line: thermodynamic model prediction from^40^ as Equation 3. Black squares: radii of SEs measured via Nanog co-localization fluorescence images from mESCs^35^; error bars show standard deviations of multiple measurements. (B) Dependence of SE radius increment ΔR on enhancer interaction strength k. Black solid line: inverse structural correlation function $g{\left(R\right)}^{-1}$; red dashed line: thermodynamic model prediction from^40^ as Equation 10. All simulations adopt parameters L = 65 kb, n = 5, and T = 297.15 K, consistent with experimental measurements.

Equation 3 reveals three principal determinants of LLPS droplet dimensions within SEs: the contour length of intra-SE chromatin L, the number of enhancer elements n, and their pairwise interaction strength k. L was determined by applying DNA-to-primary chromatin structure compaction ratios to published α-SE DNA sequence length measurements (Table 1) obtained from mouse embryonic stem cells (mESCs).^42^^,^^43^ By numerically solving both the inverse function of Equation 2 (denoted by $g{\left(R\right)}^{-1}$) and Equation 3 with these biophysical parameters, we obtained the dependence of droplet radius R on the interaction strength k. Notably, previous studies have reported that the chromatin elastic constant falls within the range of 5–1100 pN/nm, with lower compression levels corresponding to larger elastic constants^44^; therefore, our parameter settings for the elastic constant align with this range. As illustrated in Figure 2A, the theoretical model indicates that the radius R of LLPS droplet decreases with increasing interaction strength k, asymptotically approaching a constant value. To evaluate this theoretical relationship against experimental observations, we compared our predictions with droplet-size measurements reported previously.^35^ The close agreement between the experimental data and the theoretical curve provides strong support for the notion that the equilibrium radius of LLPS droplets is regulated by the interaction strength k of enhancers. And the curve derived from $g{\left(R\right)}^{-1}$ closely matches the results calculated using Equation 3. Specifically, as shown in Figure 2B, the LLPS droplet growth size ΔR follows the scaling law $\Delta R\propto k^{-1/2}$. Details of image-based size extraction, replicate measurements, and error-bar definition are provided in STAR Methods.

**Table 1:** Biophysical parameters of SEs in mESCs

Sequence Length (kb)	Number of enhancer elements	Temperature (K)	Compression ratio
L	n	T	cr
65	5	297.15	6.1

As demonstrated in Figure S4 of the Supplemental Material by Meng et al.,^36^ ΔP in Equation 9 primarily arises from elastic contributions rather than interfacial tension. Based on this finding, the pressure balance equation simplifies to an elasticity-dominated form.:

(Equation 4) $$\Delta P=\frac{3\gamma }{R}+f_{el}\left(R\right)\;\approx \;f_{el}\left(R\right)=kf\left(R\right),$$

where k quantifies the enhancer element interaction strength, and $f\left(R\right)=f_{el}\left(R\right)/k$ defines a function that increases monotonically with the droplet radius R.

Implementing the biophysical parameters from Table 1 into our model reveals the functional relationship:

(Equation 5) $$f\left(R\right)≃0.003R-0.82.$$

**Figure 3:**
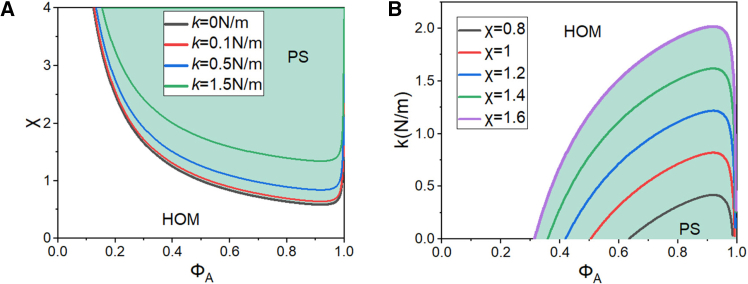
Phase diagrams of enhancer-mediated liquid-liquid phase separation (A) Phase boundaries in the $\left(\phi_{\text{A}},\chi \right)$ plane under varying enhancer interaction strengths k. (B) Phase separation thresholds in the $\left(\phi_{\text{A}},k\right)$ plane for different Flory-Huggins parameters χ. Curves demarcate homogeneous (HOM) and phase-separated (PS) regimes. Molecular volumes: $v_{A}=3.7\times 10^{-28}\;m^{3}$ (component A), $v_{B}=4.8\times 10^{-26}\;m^{3}$ (component B).

To highlight the role of the interaction strength k between enhancers, the average of f(R) over its domain is calculated through Equation 5, yielding $f\left(R\right)\approx 0.489k$. Combining this result with Equation 8 allows the calculation of the phase diagram for LLPS mediated by enhancer element interactions, as shown in Figure 3. This computational framework quantitatively links interaction strength k to the thermodynamic stability of LLPS droplets.

According to the Flory-Huggins theory, the critical value of the Flory-Huggins interaction parameter χ for LLPS is given by^45^ $\chi_{c}=1/2+1/\sqrt{N}+1/\left(2N\right)$, where χ is a dimensionless parameter that quantifies the energy of interaction between solvent and solute molecules—a higher value indicates stronger mutual repulsion—and N is the volume ratio between the two solvent species. For $\chi >\chi_{c}$, the homogeneous state becomes unstable over a range of $\phi_{A}$ values, leading to phase separation. As shown in Figure 3, the presence of enhancer element interaction forces moves the binodal line upward in the small-$\phi_{A}$ region. And shifts the critical concentration $\phi_{Ac}$ for phase separation to higher values, while the critical parameter $\chi_{c}$ increases with the interaction strength k. These results demonstrate that the synergistic interactions between enhancer elements modulate both the thermodynamic driving forces and the critical concentration required for LLPS droplet formation.

### Enhancer element size tunes droplet radius and LLPS phase diagram

TFs, together with cohesin and other co-factors, remodel chromatin topology to mediate three-dimensional chromatin architecture and long-range loop formation.^46^ The 5–20 bp motifs within TFBSs are essential for recruiting TFs to enhancers.^47^^,^^48^^,^^49^^,^^50^ Furthermore, recent studies have demonstrated that the flanking sequences of TFBSs play important regulatory roles in three-dimensional chromatin architecture, long-range interactions, and transcription.^51^ While chromatin architecture establishment and maintenance operate through transcription-independent mechanisms,^52^ the physical role of enhancer element size m in modulating the intra-droplet critical concentration for LLPS and droplet growth remains unexplored.

**Figure 4:**
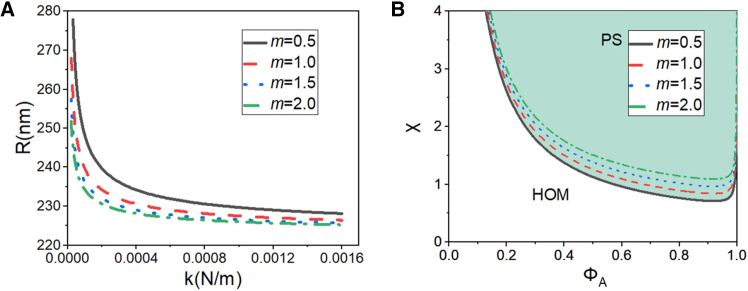
Enhancer-size dependence of SE radius and LLPS phase behavior (A) Radius of SEs regulated by enhancer interaction strength k and enhancer element size m. (B) Phase diagrams of enhancer-size-regulated LLPS. HOM: homogeneous state; PS: phase-separated state. Molecular volumes: $v_{A}=3.7\times 10^{-28}\;m^{3}$, $v_{B}=4.8\times 10^{-26}\;m^{3}$.

Figure 4A displays the dependence of LLPS droplet radius R on enhancer interaction strength k for different enhancer element sizes m, which were systematically incorporated into the α-SE structural parameters. The curves were generated by numerically solving $g{\left(R\right)}^{-1}$ using the biophysical parameters listed in Table 1.

Figure 4A demonstrates an inverse relationship between enhancer element size m and the radius of LLPS droplets R at fixed interaction strength k. This size-dependent regulation shows heightened sensitivity under weak interaction conditions. However, the sensitivity progressively diminishes as k increases, ultimately leading to convergence of droplet radii toward a homeostatic size. The parametrization of Equation 1 with varying *m* values combined with the established mean-field approximation from Equation 8 and Equation 4 yields m-dependent phase diagrams, as shown in Figure 4B.

As shown in Figure 4B, larger enhancer elements (with increased m) exhibit stronger resistance to phase separation. Figure 4B indicates that as the size m of the enhancer element increases, the binodal curve shifts upward and to the right, suggesting that the critical Flory-Huggins parameter $\chi_{c}$ and the critical concentration $\phi_{Ac}$ both increase with the size m of the enhancer element.

### Sensitivity analysis of droplet radius to chromatin length and element number

**Figure 5:**
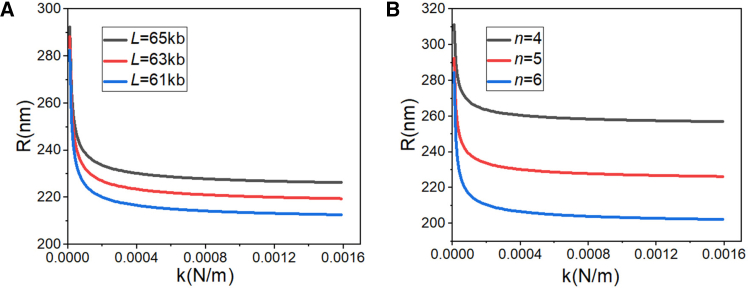
SE radius as a function of enhancer interaction strength and intra-SE chromatin architecture parameters (A) Radius of SEs regulated by enhancer interaction strength k and the total number of elements n. (B) Radius of SEs regulated by enhancer interaction strength k and the length of intra-SE chromatin L.

To investigate the sensitivity of our model, we systematically varied the chromatin length L or the number of enhancers n and computed the corresponding curves of droplet radius R as a function of the interaction strength k. All computed R values fell within the expected physical range, demonstrating the robustness of the model. As shown in Figure 5A, when n was held constant (for values, see Table 1), R exhibited a positive correlation with L. Conversely, as presented in Figure 5B, when L was fixed (for values, see Table 1), R was negatively correlated with n. Comparative analysis revealed that R is more sensitive to variations in n than to changes in L.

## Discussion

In recent years, SEs have become a research hotspot due to their unique structure and function. Meanwhile, LLPS droplets, as the core structure of SEs, has been well characterized throughout its life cycle through the competition between multivalent interactions and electrostatic interactions mediated by transcriptional RNA production.^34^ It is evident that the nuclear environment where SEs reside is highly crowded, and SEs exhibit significant differences in size and transient dynamic properties compared to other LLPS droplets in eukaryotic cells.^3^ These findings suggest the existence of a mechanical constraint that restricts the size of LLPS droplets within SEs, enabling SEs to both facilitate high-level gene expression and adapt to the crowded nuclear environment. However, the mechanism by which mechanical constraints regulate the structure of SEs has not been systematically elucidated. Therefore, this study aims to construct a kinetic model to reveal the underlying mechanism of this process.

Surprisingly, in the 2023 experimental study of α-SE by Kassouf and the Higgs team, they described a special enhancer element termed the “facilitator.” Although the study detailed the structural and functional characteristics of the facilitator, it did not elucidate its mechanism within SEs, only comparing the effects of knocking out a single facilitator versus all facilitators on gene expression.^42^ Therefore, we speculate that the facilitator may primarily function to maintain the structural stability of SEs. We model the internal chromatin structure of SEs as an open chromatin compartment that allows for the expansion of LLPS droplets while imposing physical constraints. In this framework, the interactions between facilitators and classical enhancers are abstracted as a harmonic spring force, characterizing the effective stiffness of the protein-mediated chromatin loop. This mechanical constraint restricts the growth of SEs and regulates the critical concentration of internal LLPS droplets.

The strength of this constraint is governed by the interaction strength k, the number of elements n, and the size of the elements m, while the chromatin length L defines the geometric boundary for the growth of LLPS droplets.

We incorporated the structural parameters of α-SE in mESCs into the model to evaluate the dependence of SEs' size on the interaction strength k among enhancer elements. The results indicate that the droplet radius R formed via LLPS within the SE decreases monotonically with increasing interaction strength, and the growth scale follows the scaling relation $\Delta R\propto k^{-1/2}$. The theoretical predictions are in strong agreement with experimental observations. These findings align with the dynamic behavior of polymer solutions, suggesting that the established model provides a plausible explanation for the experimental phenomena and offers a perspective for further investigation into the structure and function of SEs.

The model calculations demonstrate that the mechanical constraints of chromatin significantly alter the thermodynamic properties of phase separation by increasing both the critical concentration $\phi_{Ac}$ and the Flory-Huggins parameter $\chi_{c}$ for LLPS. Meanwhile, enhancer size m serves as a key regulatory factor: larger m values further suppress droplet growth, manifested by an increase in $\chi_{c}$ and $\phi_{Ac}$ at a fixed interaction strength k. These effects originate from the spatial confinement imposed by chromatin on LLPS droplet size and the size-dependent modulation of interactions by enhancers, collectively constituting the biophysical basis of LLPS. Further analysis of model sensitivity, based on a robust parameter analysis, revealed that increased chromatin length L promotes droplet expansion, whereas a greater number of enhancer elements n more strongly suppresses this process. These results illustrate how specific structural parameters of enhancer clusters can precisely set the radius of LLPS droplets by modulating mechanical constraints.

In 2019, the molecular process by which RNA polymerase II (Pol II), regulated by the dynamic phosphorylation of its carboxy-terminal domain (CTD), transitions from transcriptional LLPS droplets to splicing LLPS droplets was revealed.^53^ That same year, Shrinivas demonstrated through modeling and experiments that at low protein concentrations, DNA can significantly promote the nucleation of LLPS and maintain droplet stability, a process governed by factors such as TF-DNA affinity, binding site valency, and density.^54^ In 2022, Team Morin further proposed a “pre-wetting” model, suggesting that at subcritical concentrations, proteins can mediate the formation of transcriptional LLPS droplets on chromatin surfaces by leveraging their high affinity for specific DNA sequences.^55^ Collectively, these studies indicate that phase separation in biological systems exhibits marked “proactivity,” enabling local nucleation even when global concentrations are below the critical threshold. The underlying mechanism effectively increases the effective χ of the system’s components, thereby significantly lowering the concentration threshold required for phase separation to occur. Notably, whereas previous models have addressed the nucleation phase of LLPS, the present work investigates the post-nucleation growth of droplets.

It has been suggested that, although cooperative interactions may exist among individual enhancer elements within SEs, overall expression levels largely conform to an additive model.^38^ In contrast, a quantitative enhancer expression model established in drosophila in 2016 explicitly introduced transcription factor concentration as a key variable regulating enhancer activity.^39^ This study further reveals, through the construction of phase diagrams, that mechanical constraints not only suppress the growth of LLPS droplets but also significantly affect the component concentration within the droplets. Furthermore, phase diagram analysis in this study revealed that mechanical confinement not only suppresses the growth of LLPS droplets but also significantly alters their internal component concentration. This indicates that the interaction strength k among enhancer elements and their size m can cooperatively regulate the LLPS droplet state of LLPS. Specifically, mechanical confinement modulates the saturated concentration within droplets by altering the osmotic pressure between the droplets and their surrounding environment. This change in concentration ultimately governs the intensity of transcriptional bursting. Therefore, this study proposes a potential mechanism linking mechanical constraints to SE function, highlighting the important role of structural mechanical factors—beyond sequence information—in the regulation of SEs, and provides a research direction for a deeper understanding of SE regulatory logic from a structure-function relationship perspective.

### Limitations of the study

A set of simplifying assumptions is adopted to isolate the contribution of chromatin structural constraints to the growth of SE-associated droplets. Specifically, the effective rigidity of protein-mediated chromatin loops is idealized as a Hookean spring force to model interactions between enhancer elements, and LLPS droplets are assumed to be spherically symmetric. These assumptions neglect nonlinear and viscoelastic mechanical responses, time-dependent chromatin remodeling, and anisotropic morphologies that can arise under spatially heterogeneous confinement; future work should therefore incorporate anisotropic droplet geometries and explicit chromatin polymer dynamics to link loop architecture, mechanical response, and droplet morphology more realistically. In addition, the present framework emphasizes passive two-component phase separation and does not explicitly account for ATP-dependent transcriptional activity, chemical conversion, or turnover of droplet components in the multicomponent nuclear environment. In three-component active systems, droplet size can stabilize when diffusive flux from the dilute phase to the dense phase balances reaction-driven material consumption within the droplet.^56^ Under such conditions, chromatin confinement may modulate interfacial transport and diffusive supply, suggesting that the *in vivo* size of SE-associated LLPS droplets may be jointly governed by chromatin-mediated mechanical constraints and the intensity of internal active processes. Incorporating these nonequilibrium, multicomponent effects into chromatin-constrained models remains an important objective for future studies.

## Resource availability

### Lead contact

Requests for further information and resources should be directed to and will be fulfilled by the lead contact, Lirong Zhang (pyzlr@imu.edu.cn).

### Materials availability

This study did not generate new unique reagents.

### Data and code availability

• Data reported in this article will be shared by the lead contact upon request.
• All original code has been deposited to GitHub and is publicly available at https://github.com/TingheGuo/LLPS; it has also been archived on Zenodo with DOI: https://doi.org/10.5281/zenodo.18690457.
• Any additional information required to reanalyze the data reported in this article is available from the lead contact upon request.

## Acknowledgments

This work was supported by the National Natural Science Foundation of China (62462048, 61962041, 52263032) and the Natural Science Foundation of Inner Mongolia (2022QN03012).

## Author contributions

T.G.: data curation, formal analysis, investigation, and writing – original draft. N.Z.: data curation, formal analysis, and investigation. Y.L.: data curation, formal analysis, and investigation. S.H.: data curation, formal analysis, and investigation. J.L.: conceptualization, data curation, formal analysis, funding acquisition, investigation, methodology, project administration, resources, supervision, and writing – original draft. L.Z.: conceptualization, data curation, formal analysis, funding acquisition, investigation, methodology, project administration, resources, supervision, and writing – original draft.

## Declaration of interests

The authors declare no conflict of interest.

## Declaration of generative AI and AI-assisted technologies in the writing process

The authors declare that they have used generative AI, specifically GPT-5.2, to improve language and readability. After using this tool, the authors reviewed and edited the content as needed and take full responsibility for the content of the publication.

## STAR★Methods

### Key resources table

**Table:** 

REAGENT or RESOURCE	SOURCE	IDENTIFIER
**Software and algorithms**		

Mathematica 14.0,Image-Pro Plus 6.0,Origin 2021	Wolfram Research,Media Cybernetics,OriginLab Corporation	–
LLPS model code	GitHub, Zenodo	https://github.com/TingheGuo/LLPS; https://doi.org/10.5281/zenodo.18690457

### Method details

#### Model formulation

LLPS in biological systems typically involves multicomponent molecular interactions. To elucidate the mechanical constraints imposed by SEs’ peripheral chromatin architecture on LLPS dynamics, the system is modeled as a simplified binary fluid mixture comprising components A and B. LLPS leads to the formation of a droplet enriched in one fluid component, characterized by a radius R. Under the condition of mass conservation for component A, the relationship between the volume fraction of A in the droplet phase $\left(\phi_{A}^{d}\right)$ and that in the bulk phase $\left(\phi_{A}^{b}\right)$ obeys the following conservation relationship:

(Equation 6) $$v\phi_{A}^{d}+\left(1-v\right)\phi_{A}^{b}=\phi_{A}^{0},$$

where $\phi_{A}^{0}$ denotes the homogeneous volume fraction of component A before phase separation occurs. The total free energy density of the system is governed by:

(Equation 7) $$\begin{array}{cc} F\left(\phi_{A}^{d},\phi_{A}^{b},v,R\right) & =vf_{\text{mix}}\left(\phi_{A}^{d}\right)+\left(1-v\right)f_{\text{mix}}\left(\phi_{A}^{b}\right)\\ & -\xi \left[v\phi_{A}^{d}+\left(1-v\right)\phi_{A}^{b}-\phi_{A}^{0}\right]\\ & +3v\frac{\gamma }{R}+vf_{\text{el}}\left(R\right). \end{array}$$

The first two terms describe the Flory-Huggins mixing free energy density in the droplet $\phi_{A}^{d}$ and bulk $\phi_{A}^{b}$ phases,where v is the volume of the droplet phase. The $f_{\text{mix}}\left(\phi_{A}\right)$ follows the standard form^57^:

(Equation 8) $$\begin{array}{ll} f_{\mathrm{mix}}\left(\phi_{A}\right) & =\left(k_{B}T/v_{A}\right)\left[\chi \phi_{A}\left(1-\phi_{A}\right)\right)\\ & \left(+\phi_{A}\ln\left(\phi_{A}\right)+\left(1/N\right)\left(1-\phi_{A}\right)\ln\left(1-\phi_{A}\right)\right], \end{array}$$

the parameter N, defined as the volume ratio between the two solvent species. The third term in Equation 7 describes the conservation of chemical potential under the constraint imposed by Equation 6, where ξ acts as the Lagrange multiplier.^36^ The fourth term corresponds to the interfacial energy density of the droplet, with γ denoting the interfacial tension coefficient. Finally, the elastic energy $f_{\text{el}}\left(R\right)$, arising from SE -associated chromatin structural deformation, imposes mechanical resistance that restricts droplet growth.

Compared to TEs, SEs exhibit a clustered architecture of multiple enhancer elements, as illustrated in Figure 1A. The effective stiffness of protein-mediated chromatin loops was abstracted as the interaction strength between enhancer elements. As shown in Figure 1B, the interaction force between two enhancer elements is modeled using the linear elastic force F=−k⋅x from Hooke’s law, where k quantifies the interaction strength, and x represents the spatial separation between elements. The geometrical distance is defined as $x=\sqrt{2r^{2}\left(1-\cos\beta \right)}$ where r is the radial distance of enhancer elements from the droplet centroid, β is the angular separation between elements, l denotes the enhancer penetration depth, and R=r+l is the radius of the LLPS droplet.

Recent experimental studies by Kassouf, Higgs, and colleagues^42^ on the mouse α-globin super-enhancer (α-SE) revealed that SEs comprise two functionally distinct components: classical enhancers, which directly activate or enhance target gene expression, and facilitators, which lack intrinsic enhancer activity but are essential for enabling classical enhancers to achieve full transcriptional activation. This bipartite architecture underscores the cooperative nature of SEs. Building on the experimental findings of Kassouf and Higgs,^42^ our model incorporates functional asymmetry between enhancer elements: classical enhancer elements exhibit preferential interactions with multiple facilitator elements rather than engaging in uniform pairwise coupling. The surrounding chromatin network imposes constraints resembling those of elastic media. Furthermore, the deformation into an oblate spheroid results from the anisotropic confinement of the one-dimensional chromatin polymer—primarily restricting expansion along the chain axis—and the resistance to deformation offered by the LLPS droplet’s Maxwell fluid-like properties.^58^^,^^59^^,^^60^ In addition to LLPS driven by elastic interactions, alternative mechanisms can govern the formation of size-limited biomolecular LLPS droplets. These include competing short-range attractive (e.g., van der Waals) and long-range repulsive (e.g., Coulombic) interactions between phase-separating components,^61^^,^^62^ as well as finite-component effects arising from molecular stoichiometric constraints.^7^^,^^63^ Based on the findings of Michael R. O’Dwyer et al. in 2025 regarding the regulatory functions of TFBS flanking sequences in gene expression, such as mediating long-range interactions, we defined the enhancer element size m, a parameter scaling its relative size, to include the TFBSs and their flanking regions.^51^

These complementary mechanisms may operate synergistically with chromatin elasticity to regulate SE architecture. The consistent pressure difference between LLPS droplets and the bulk phase establishes a mechanical equilibrium, allowing the dimensional constraints of droplets in orthogonal directions to be reformulated as effective mechanical constraints through equivalent variational formulations. Through modeling the effective volume of elastic constraint, as a sphere of radius R (see STAR Methods, method details, “effects of constraints on LLPS droplet morphology”), we derive the elastic energy density expression is Equation 1. In Equation 1, β=2πy/n, y represents the relative displacement between two enhancer elements, k is the enhancer-facilitator interaction strength, m represents the element sizes, n denotes the total number of elements, L is the length of intra-SE chromatin, R corresponds to the radius of the LLPS droplet, and Δh is defined as the microscopic effective width (substantially smaller than the droplet size) over which the SE-associated chromatin structure acts. It is set to unity for simplicity in subsequent discussions. These parameters collectively define the chromatin-mediated mechanical constraints.

Consistent with the findings of Meng et al., and as shown in Figures 1C and 1D, LLPS in elastic networks is strongly modulated by elasticity, where droplet size and distribution are dictated by the interplay of surface tension and elastic deformation. It follows that under thermodynamic equilibrium, the chemical potential of component A is equalized between the LLPS droplet and bulk phase$\left(\mu_{A}^{d}=\mu_{A}^{b}\right)$. Consequently, the pressure difference satisfies the mechanical equilibrium condition^36^:

(Equation 9) $$\Delta P=\Pi \left(\phi_{A}^{d}\right)-\Pi \left(\phi_{A}^{b}\right)=\frac{3\gamma }{R}+f_{el},$$

where $\Pi \left(\phi_{A}^{b}\right)$ and $\Pi \left(\phi_{A}^{d}\right)$ denote the osmotic pressures in the bulk phase and droplet phase, respectively. This equation balances three mechanical contributions: the osmotic pressure differential, the surface tension energy, and the chromatin-mediated elastic constraints arising from enhancer element interactions. To discuss the relationship between the size of LLPS droplets and the free energy density, we analyze the derivative of the elastic free energy density $f_{\text{el}}$ with respect to R. This reveals the competition between surface tension and chromatin-mediated elastic forces is Equation 2. In Equation 2, β=2πy/n,This equality demonstrates how surface tension counterbalances elastic forces to determine droplet size. The function g(R) encapsulates enhancer element interactions through the parameters k, m, n, and L defined in Equation 1.

In an elastic network, the mesh size $R_{0}$ is related to the shear modulus G through $R_{0}≃{\left(k_{\text{B}}T/G\right)}^{1/3}$.^64^ Analogously, for an ideal Gaussian chain representing elastic behavior, the coil size $R_{0}$ is related to the elastic constant K by $R_{0}≅{\left(k_{\text{B}}T/K\right)}^{1/2}$,^40^^,^^41^ where $k_{\text{B}}$ is the Boltzmann constant and T denotes temperature. Furthermore, the equilibrium size of a LLPS droplet within an elastic network satisfies, where $\lambda_{m}$ represents the maximal stretching limit of the chromatin chain.^36^

In our model, since interactions exist only between the enhancer elements, the coil size corresponds to the distance r from the center to an enhancer element (illustrated in Figure 1), following the relation $r≃{\left(k_{\text{B}}T/k\right)}^{1/2}$. Therefore, the equilibrium distance of an enhancer element from the center of the phase-separated droplet is given by:

(Equation 10) $$\Delta R=\Delta R_{\max}\cdot r=\frac{n+\pi }{\pi }\cdot {\left(\frac{k_{\text{B}}T}{k}\right)}^{\frac{1}{2}}$$

where $\Delta R_{\max}$ denotes the maximum change in the droplet radius. Because the chromatin chain is not subject to elastic constraints in our model, the droplet possesses an initial radius $R_{0}$ (i.e., the maximum radius in the absence of elastic constraints) under the critical condition where the distance between enhancer elements x=0. Consequently, the overall equilibrium radius of the droplet in Equation 3.

#### Derivation of elastic energy density

Compared to TEs, SEs exhibit a clustered architecture of multiple enhancer elements, as illustrated in Figure 1A. The effective stiffness of protein-mediated chromatin loops was abstracted as the interaction strength between enhancer elements. As shown in Figure 1B, the interaction force between two enhancer elements is:

(Equation 11) F=−k⋅x

The corresponding linear elastic energy is:

(Equation 12) $$F_{\text{el}}=\frac{1}{2}mkx^{2}$$

where $x=\sqrt{2r^{2}\left(1-\cos\beta \right)}$, r is the radial distance of enhancer elements from the droplet centroid, β is the angular separation between elements. l denotes the enhancer penetration depth and R=r+l is the radius of the LLPS droplet, So:

Equation (13) $$l=\frac{L-2\pi R}{2n}$$

(Equation 14) $$\begin{array}{cc} F_{el} & =\frac{1}{2}\overset{n-1}{\underset{y=1}{\sum }}km2r^{2}\left(1-\cos\beta \right)\\ & =\frac{1}{2}\overset{n-1}{\underset{y=1}{\sum }}km2\left(1-\cos\beta \right){\left(R-l\right)}^{2}\\ & =\frac{1}{2}\overset{n-1}{\underset{y=1}{\sum }}km2\left[1-\cos\left(\frac{2\pi y}{n}\right)\right]{\left(R-l\right)}^{2}\\ & =\frac{1}{2}km2\left[1-\cos\left(\frac{2\pi y}{n}\right)\right]{\left(R-\frac{L-2\pi R}{2n}\right)}^{2} \end{array}$$

The term $F_{el}$ denotes the elastic free energy arising from inter-enhancer interactions, which effectively acts within a volume, $v_{0}$. To investigate the effect of mechanical constraints on the size of LLPS droplets, we generalize this concept by modeling $v_{0}$ as a sphere with a radius R.

(Equation 15) $$\begin{array}{cc} E & =∭\frac{F_{el}}{v_{0}}dv\\ & =\overset{n-1}{\underset{y=1}{\sum }}\frac{1}{2}\frac{km\left(1-\cos\beta \right)}{\Delta h}∭\frac{{\left(L-2\left(n+\pi \right)R\right)}^{2}}{4n^{2}\pi R}dv\\ & =\overset{n-1}{\underset{y=1}{\sum }}\frac{1}{2}\frac{km\left(1-\cos\beta \right)}{\Delta h}\frac{{\left(2\left(n+\pi \right)R-L\right)}^{3}}{3n^{2}\left(n+\pi \right)} \end{array}$$

Here, Δh denotes the microscopic effective width of the chromatin structure’s action, so the elastic energy density expression and the elastic energy density is expressed as Equation 1. Since Δh is substantially smaller than the droplet size, we normalize it to unity to simplify the subsequent discussion.

#### Effects of constraints on LLPS droplet morphology

Extensive studies have shown that biomolecular condensates formed through phase separation *in vivo* exhibit Maxwell viscoelasticity, enabling them to resist external deformation.^58^^,^^59^^,^^60^ Consequently, under specific elastic constraints, such as those imposed by chromatin structures, the droplets can transition from a spherical to an oblate spheroidal shape. Within the framework of continuum mechanics, the elastic free energy of the system is given by^65^:

(Equation 16) $$E=G{\left(u_{ik}-\frac{1}{3}\delta_{ik}u_{ll}\right)}^{2}+\frac{\mu }{2}u_{ll}^{2}$$

where the first term on the right-hand side represents the shear deformation energy, and the second term corresponds to the volumetric deformation energy. Here, $u_{ik}$ is a component of the strain tensor, $u_{ll}$ is its trace indicating the relative volume change, G is the shear modulus, and μ is the bulk modulus.

When analyzing the effect of elastic constraints on the size of LLPS droplets, the chromatin-imposed constraint induces deformation along the normal direction, leading to an increased curvature. This deformation balances the internal pressure of the droplet without altering the length of its minor semi-axis. Based on this conceptual framework, we map the effect of the constraint onto an effective spherical cavity with a radius equal to the minor semi-axis of the oblate spheroid. The effect of the normal constraint is thus compensated by the surface tension arising from the curvature change. This treatment significantly simplifies the model by neglecting shear deformation and treating the droplet as a sphere defined by its minor semi-axis. This allows us to focus on how the uniform compression term regulates the droplet size, thereby elucidating the role of the elastic network in controlling condensate dimensions.

#### Contributions to the pressure difference

The Macleod empirical relation expresses the surface tension, γ, as a function of the density difference between the two components^66^^,^^67^:

(Equation 17) $$\gamma =B{\left(\rho_{1}-\rho_{2}\right)}^{4}$$

where B is a constant. In liquid-liquid systems, pressure is unable to significantly alter the density difference $\left(\rho_{1}-\rho_{2}\right)$. Hence, variations in surface tension are negligible under modest pressure changes. For this reason, when analyzing the influence of mechanical constraints on the critical conditions of phase separation, the primary contribution arises from the elastic term, while the role of interfacial tension can be neglected.

### Quantification and statistical analysis

#### Published data extraction

Droplet-size measurements used for quantitative comparison with the model were extracted from previously published microscopy images (Figure 1).^35^ Droplet boundaries were delineated in Image-Pro Plus 6.0 using a semi-automated procedure with manual inspection. For each droplet, the minor-axis radius was taken as the characteristic size. This choice reflects the possibility of anisotropic droplet shapes under chromatin-mediated confinement; the minor axis provides a conservative estimate of the droplet core dimension and is consistent with the spherical-droplet approximation used in the theoretical framework. Pixel distances were converted to physical units (nm) using the scale bar provided in the published figure. Each droplet was independently outlined and measured three times. Reported data points correspond to the mean of the three measurements, and error bars represent the standard deviation across repeats. Model–data consistency was assessed by evaluating whether the experimentally measured distribution of droplet minor-axis radii falls within the theoretically predicted range of equilibrium radii. Based on this criterion, we inferred the enhancer-element interaction strength required to stabilize a droplet-size distribution consistent with the experimental observations, interpreted here as the effective stiffness of protein-mediated chromatin looping.
