# Supplementary material for: Elastic enhancer network tunes equilibrium thermodynamics of liquid liquid phase separation in super enhancers
Source: iScience. 2026 Mar 4;29(4):115152. doi: 10.1016/j.isci.2026.115152 (PMC13049525; doi:10.1016/j.isci.2026.115152)
Supplement: Document S1. Table S1 [file mmc1.pdf]

**iScience, Volume *29***

## Supplemental information

# Elastic enhancer network tunes equilibrium thermodynamics of liquid liquid phase separation in super enhancers

**Tinghe Guo, Nan Zhang, Yannan Li, Shaoqian Hao, Junjie Liu, and Lirong Zhang**

# Table S1

**Table S1.** Parameter table

| Parameter | Physical interpretation | Dimensions or value ranges | Parameter values |
|---|---|---|---|
| $k_B$ | the Boltzmann constant | J/K | $1.38 \times 10^{-23}$ |
| $T$ | temperature | K | 297.15 |
| $v$ | the volume of the droplet phase | $nm^3$ | – |
| $v_A$ | the molecular volume of component A | $m^3$ | $3.7 \times 10^{-28}$ |
| $v_B$ | the molecular volume of component B | $m^3$ | $4.8 \times 10^{-26}$ |
| $N$ | the volume ratio between the two solvent species | – | 129.73 |
| $\phi_A^0$ | the homogeneous volume fraction of component A | – | – |
| $\phi_A^d$ | the volume fraction of A in the droplet phase | – | – |
| $\phi_A^b$ | the volume fraction of A in the bulk phase | – | – |
| $\chi$ | Flory-Huggins parameters | – | – |
| $\chi_c$ | the critical value of the Flory-Huggins interaction parameter | – | – |
| $\gamma$ | the interfacial tension coefficient | N/m | – |
| $R$ | the droplet radius | nm | – |
| $x$ | the spatial separation between elements | nm | – |
| $r$ | the radial distance of enhancer elements from the droplet centroid | nm | – |
| $l$ | the enhancer penetration depth | nm | – |
| $\beta$ | the angular separation between elements | – | – |
| $k$ | the enhancer element interaction strength | N/m | – |
| $m$ | the element sizes (the TFBSs and their flanking regions) | nm | – |
| $v_0$ | actual volume acted upon by the elastic constraints originating from the SE-associated chromatin structure | $nm^3$ | – |
| $F_{el}$ | elastic free energy arising from the SE-associated chromatin structure | J | – |
| $E$ | the elastic free energy of the system | J | – |
| $\Delta h$ | the effective width over which the SE-associated chromatin structure actually acts on the droplet | nm | – |
| $L$ | the length of intra-SE chromatin (SE-associated sub-TAD length) | kb | 65 |
| $\Delta p$ | the pressure difference across the interface of a phase-separated droplet | $N/m^2$ | – |
| $\Pi(\phi_A^d)$ | the osmotic pressures in the droplet phase | – | – |
| $\Pi(\phi_A^b)$ | the osmotic pressures in the bulk phase | – | – |
| $n$ | the total number of elements | – | 5 |
| $f_{el}$ | the elastic free energy density | $J/m^3$ | – |

| Parameter | Physical interpretation | Dimensions or value ranges | Parameter values |
|---|---|---|---|
| $c_r$ | the compaction ratio from the DNA sequence length to the primary chromatin fold | 6–7 | 6.1 |
| $F\left(\phi_A^d, \phi_A^b, \nu, R\right)$ | the total free energy of the system | J | – |
| $f_{\text{mix}}(\phi_A)$ | the Flory-Huggins mixing free energy density of component A | J/m$^3$ | – |
| $f_{\text{mix}}(\phi_A^d)$ | the Flory-Huggins mixing free energy density in the droplet phase | J/m$^3$ | – |
| $f_{\text{mix}}(\phi_A^b)$ | the Flory-Huggins mixing free energy density in the bulk phase | J/m$^3$ | – |
| $\xi$ | the Lagrange multiplier | – | – |
| $F$ | the interaction force between two enhancer elements | N | – |
| $R_0$ | the droplet possesses an initial radius (the coil size) | nm | 222.496 |
| $K$ | the elastic constant | N/m | – |
| $\Delta R_{\text{max}}$ | the maximum change in the droplet radius | – | 0.38587 |
| $\Delta R$ | the condensate growth size | nm | – |
| $G$ | the shear modulus | – | – |
| $\mu$ | the bulk modulus | – | – |
| $u_{ll}$ | the trace indicating the relative volume change | – | – |
| $u_{ik}$ | the component of the strain tensor | – | – |
